# New Insights into the Hourly Manure Coverage Proportion on the Manure Belt in a Typical Layer House for Accurate Ammonia Emission Modeling

**DOI:** 10.3390/ani11082433

**Published:** 2021-08-18

**Authors:** Li Yang, Chaowu Yang, Chenming Hu, Chunlin Yu, Siyang Liu, Shiliang Zhu, Mohan Qiu, Hongqiang Zhu, Lingzhi Xie, Longhuan Du

**Affiliations:** 1 Sichuan Animal Science Academy, Chengdu 610066, China; yangli_sasa@163.com (L.Y.); cwyang@foxmail.com (C.Y.); huchenming@126.com (C.H.); yuchunlin1984@sina.com (C.Y.); uniyaliu@163.com (S.L.); zhushiliang1994@163.com (S.Z.); mohan.qiu@163.com (M.Q.); 2 Sichuan Shengxing Intelligent Technology Company, Chengdu 611436, China; shxgroupzhu@163.com; 3 Department of Mechanics, College of Architecture and Environment, Wangjiang Compus, Sichuan University, Chengdu 610065, China; xielingzhi@scu.edu.cn

**Keywords:** manure area, manure coverage proportion, environment control, ammonia emission, layer house

## Abstract

**Simple Summary:**

Hourly manure coverage proportion and area on the manure belt are key parameters for estimating ammonia emissions in poultry houses in order to provide environmental control suggestions and achieve the goals of precision poultry farming. In this study, experimental measurements were performed, and binary images were applied to provide new insights into the projected hourly manure coverage area on the manure belt at different layer hen ages. It was demonstrated that manure coverage proportion and area measured at different laying hen ages showed similar trends and values with four distinct stages within 48 h. In addition, statistical analyses found no significant correlation between the hourly increment of manure weight and the hourly increment of manure coverage proportion. The results from the present study are expected to serve as a fundamental input parameter for ammonia emission modeling to more accurately simulate the hourly indoor environment and provide effective mitigation strategies.

**Abstract:**

The main advantage of having livestock, for example, the laying hens, in a controlled environment is that the optimum growth conditions can be achieved with accuracy. The indoor air temperature, humidity, gases concentration, etc., would significantly affect the animal performance, thus should be maintained within an acceptable range. In order to achieve the goals of precision poultry farming, various models have been developed by researchers all over the world to estimate the hourly indoor environmental parameters so as to provide decision suggestions. However, a key parameter of hourly manure area in the poultry house was missing in the literature to predict the ammonia emission using the recently developed mechanistic model. Therefore, in order to fill the gap of the understanding of hourly manure coverage proportion and area on the manure belt, experimental measurements were performed in the present study using laying hens from 10 weeks age to 30 weeks age. For each test, six polypropylene (pp) plates were applied to collect the manure dropped by the birds every hour, and photographs of the plates were taken at the same time using a pre-fixed camera. Binary images were then produced based on the color pictures to determine the object coverage proportion. It was demonstrated that for laying hens of stocking density around 14 birds/m^2^, the manure coverage proportion at the 24th hour after the most recent manure removal was about 60%, while the value was approximately 82% at the 48th hour. Meanwhile, for laying hens at different ages, the hourly increment of manure coverage proportion showed a similar pattern with four distinct stages within 48 h. The statistical analyses demonstrated no significant correlation between the hourly increment of manure weight and the hourly increment of manure coverage proportion. Finally, prediction models for estimating the hourly manure coverage proportion on the manure belt in typical laying hen houses were provided.

## 1. Introduction

In recent decades, the intensification of poultry production in China has contributed to ensuring increasing demand for domestic livestock. Small farms with traditional systems have been replaced by controlled environment housings. Poultry production in an enclosed environment with high stocking density becomes an important source of ammonia (${\mathrm{NH}}_{3}$) emission, which has a number of negative effects not only on the indoor air quality but also on the ecosystem [1,2,3]. For typical poultry production, the ammonia originates from the decomposition of nitrogen content in manure and the production and emission of the ${\mathrm{NH}}_{3}$ are a result of complex biological, physical, and chemical processes [4,5]. Moreover, various factors, including ventilation rate, temperature, humidity, stocking density, management, etc., would affect the indoor ammonia concentration and emissions [6,7,8].

The ammonia concentration in controlled environment housings should be kept within an acceptable range [9,10] since a high concentration of ${\mathrm{NH}}_{3}$ had been demonstrated to be associated with health risks for both birds and exposed workers [11,12]. Therefore, it is crucial to understand and model the ammonia emissions in poultry houses so as to provide information to develop appropriate mitigation and management strategies. Much work had been done to predict ammonia release from manure [13,14,15,16,17], and several types of models were developed in the literature, including statistical models [18,19], balance models [20,21], and process-based models [22,23]. More recently, Tong et al. [24] developed a mechanistic model, which was based on the fundamental understanding of physical and biochemical processes of ammonia emissions from manure, to estimate the ${\mathrm{NH}}_{3}$ emissions rate (ER, mg $m^{-2}h^{-1}$) from laying hen manure. Information including manure pH, manure moisture content (MC), air velocity, air temperature, etc., were required for the model, and readers could refer to the original paper for more detailed information. The total ammonia emissions ($M_{\mathrm{NH}3},{\;\mathrm{mg}\;h}^{-1}$) could then be calculated by $M_{\mathrm{NH}3}=\mathrm{ER}\times A_{s}$, where $A_{s}$ was the manure surface area, $m^{2}$.

Knowing the parameter of $A_{s}$, the above mechanistic model could be effectively incorporated into many recently developed thermodynamic models [25,26,27], which were used to predict the indoor hourly environmental parameters, including ammonia emissions, and provide decision suggestions in order to achieve the goal of precision poultry farming. Nevertheless, a review of published literature demonstrates that very limited information is available relating to $A_{s}$ for laying hen production. Considering the difficulty for accurately measuring the hourly $A_{s}$, researchers in the literature applied the manure projected area, $A_{p}$, on the manure belt to approximate the $A_{s}$. According to a recent study performed by Tong et al. [28], the manure coverage proportion (MCP) on the manure belt per day, or more specifically, the coverage proportion of projected manure area on the manure belt per day, was estimated by the equation $\mathrm{MCP}=\min\left\{\frac{1}{3}+\frac{d-1}{3},\;1\right\}$, where d was the number of days after manure removal, min{a,b} equaled the smaller value between a and b. Based on the above equation, the daily manure coverage proportion was estimated to be ${\mathrm{MCP}}_{\mathrm{day}1}=33.3\%,{\;\mathrm{MCP}}_{\mathrm{day}2}=66.7\%,{\mathrm{MCP}}_{\mathrm{day}3}=100\%$. Unfortunately, to the best of the authors’ knowledge, there is no hourly data of manure coverage proportion or $A_{s}$ available, which could be directly applied for the thermodynamic models for predicting hourly ammonia emissions.

Therefore, this study aimed to fill the research gap by providing new insights into the hourly manure coverage proportion and manure area on the manure belt in a typical layer house. The weekly manure pH and MC was also measured, which were important information for estimating ${\mathrm{NH}}_{3}$ emissions. Although it is noted that the hourly manure coverage proportion on the manure belt might, to some extent, be affected by diet, species, stocking density, etc., the results from the present study are expected to serve as a fundamental input parameter for thermodynamic models to more accurately simulate the hourly indoor environment and provide effective management strategies.

## 2. Materials and Methods

### 2.1. The Layer House and the Birds

The experimental measurements were conducted in an experimental-oriented manure belt layer house in Chengdu, Sichuan province. The dimensions of the house were length, 40 m, width, 9.2 m, height, 2.5 m. Tunnel ventilation is applied with evaporative cooling systems in the house, and more details about the building could be found in previous studies [29,30]. In the house, there were 4 rows of animal-occupied zone. Each row had 3 tiers of cages raising approximately 3500 birds of the parent stock of the local species characterized by partridge-like plumage and dark-shanks. A total of 8 birds were kept in each cage with a size of width 660 mm and length 860 mm, resulting in a stocking density of approximately $14\;\mathrm{birds}/m^{2}$.

### 2.2. Manure Collection

**Figure 1:**
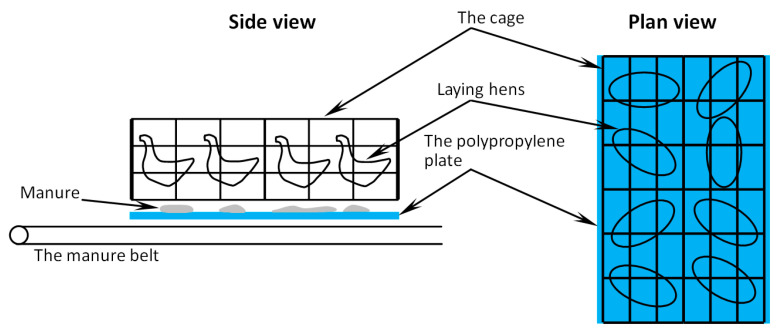
Schematic drawing of the size and placement of the polypropylene plate. The plate length is 860 mm, and the plate width is 680 mm.

Pure white polypropylene (pp) plates, which had the same width of the manure belt, 680 mm (slightly larger than the width of the cage) and a length of 860 mm (equal to the length of the cage), were hung above the manure belt in order to collect the manure dropped from the birds as schematically drawn in Figure 1. The polypropylene plates were weighted every hour so as to calculate the updated weight of the manure, and plan-view photographs of the plates with manure were also taken at the same time to determine the updated manure coverage proportion, which would be detailed in Section 2.3. In this study, the measurement campaign was conducted once a week, starting from the laying hen 10 weeks age to 30 weeks age. Meanwhile, for each measurement campaign, 6 polypropylene plates were applied and placed randomly in the poultry house providing enough data (manure produced by 48 laying hens) to calculate the hourly average values. In each week, the test began at 5 am in the morning (lights on) and lasted for 48 h (2 days). Detailed information on the measurement campaign is summarized in Table 1 below.

### 2.3. Determination of the Manure Coverage Proportion (MCP) and Area

**Figure 2:**
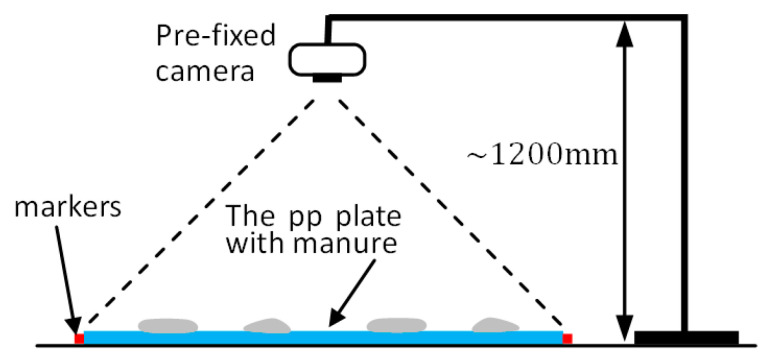
Schematic drawing of the pre-fixed camera.

To investigate the hourly manure coverage proportion (MCP) and area on the manure belt, the six polypropylene plates were moved to a pre-marked area one by one every hour to have the photographs taken by a pre-fixed camera (Figure 2). Special attention was paid when transferring the plates so as to reduce the movement of manure on the plates, which was inevitable given the fact that the manure was not ‘fixed’ on the plates. The camera lens was set perpendicular to the surface of the plate, ensuring that all the pictures were taken at the same position, height, orientation, and resolution in order to minimize the experimental error. In the present study, the resolution of the photographs was determined to be 4032×3024 pixels, which was demonstrated to be enough for the following study as pictures with more pixels did not show any significant difference in terms of the results of image processing.

**Figure 3:**
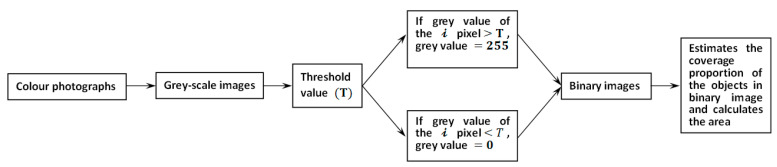
The flowchart for image processing.

To be more specific, the manure area investigated in this study was the projected area of the manure on the manure belt. The starting point of how to calculate the projected area from a picture is to estimate the manure coverage proportion in a binary image. As long as the coverage proportion could be determined, the manure area and hourly area increment could be easily calculated since the area of the background polypropylene plate is known. Therefore, the color photographs were firstly turned into gray-scale images in Matlab, and a threshold value, T=200, was used to check the gray value of each pixel in order to produce binary images, namely, a gray value smaller than 200 would be set to 0 (black) while gray value larger than 200 would be set to 255 (white). Finally, the objects coverage proportion (γ) in the binary image could be easily determined by $\gamma =\frac{\mathrm{number}\;\mathrm{of}\;\mathrm{pixels}\;\mathrm{with}\;\mathrm{gray}\;\mathrm{value}=0}{\mathrm{number}\;\mathrm{of}\;\mathrm{total}\;\mathrm{pixels}}\times 100\%$ and the area could be calculated at the same time. A flowchart is provided in Figure 3 to show the detailed image processes.

**Figure 4:**
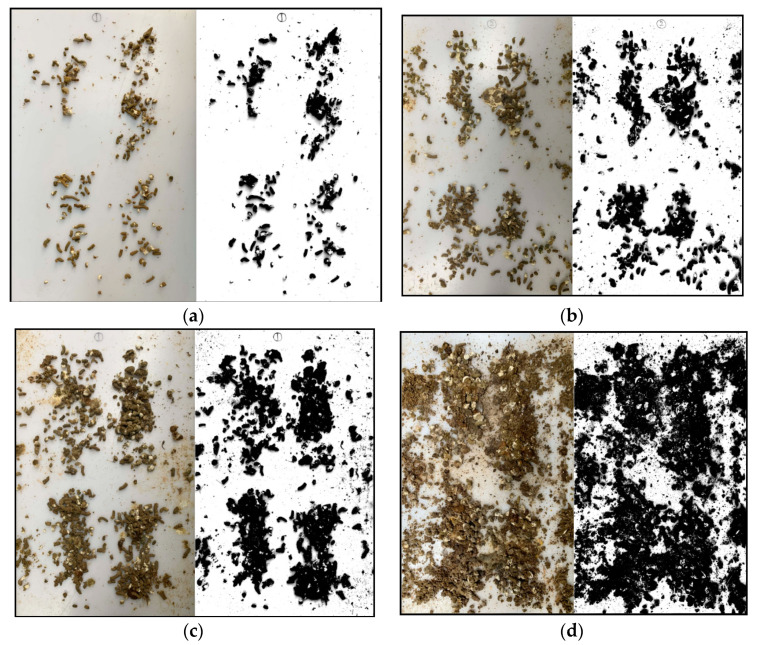
Examples of original photographs (left) and binary images (right). The photographs were taken at the (**a**) 3rd hour, (**b**) 8th hour, (**c**) 11th hour, and (**d**) 30th hour after the start of the experiment. The corresponding objects coverage proportion for (**a**–**c**) and (**d**) is 11.76%, 25.82%, 36.01%, and 68.63%, respectively.

Examples of comparisons between original color photographs and binary images are illustrated in Figure 4. As it can be clearly seen from the four pictures in Figure 4, the binary images are capable of replicating almost all details of the manure in color photographs taken at different stages of the experiment, showing the correct position and size. The corresponding manure coverage proportion for Figure 4a–d is calculated to be 11.76%, 25.82%, 36.01%, and 68.63%, respectively, and the corresponding manure area is $0.069{\;m}^{2},\;0.151{\;m}^{2},\;0.211{\;m}^{2},\;\mathrm{and}\;0.401{\;m}^{2}$, respectively. Furthermore, the limited white urate on the manure would, to some extent, affect the accuracy of coverage proportion calculated by binary images, and the maximum discrepancy was investigated to be up to approximately ±3.3% of the estimated coverage proportion value γ.

### 2.4. Manure pH, Moisture Content (MC), and Lighting

For each week during the experiment period, the manure was sampled randomly from multiple locations in the house within 3 h after manure had been dropped by the birds. The samples were then properly stored in sealed bags and transported in a timely manner to a quality-certified laboratory for determining the pH and MC. Moreover, from the laying hen 10 weeks age to 30 weeks age, the lighting program was modified regularly to achieve optimal reproductive performance through appropriate illumination and photostimulation at the appropriate age and body weight. A detailed lighting schedule for the local species is provided in Table 2.

## 3. Results and Discussion

### 3.1. Manure Weight

**Figure 5:**
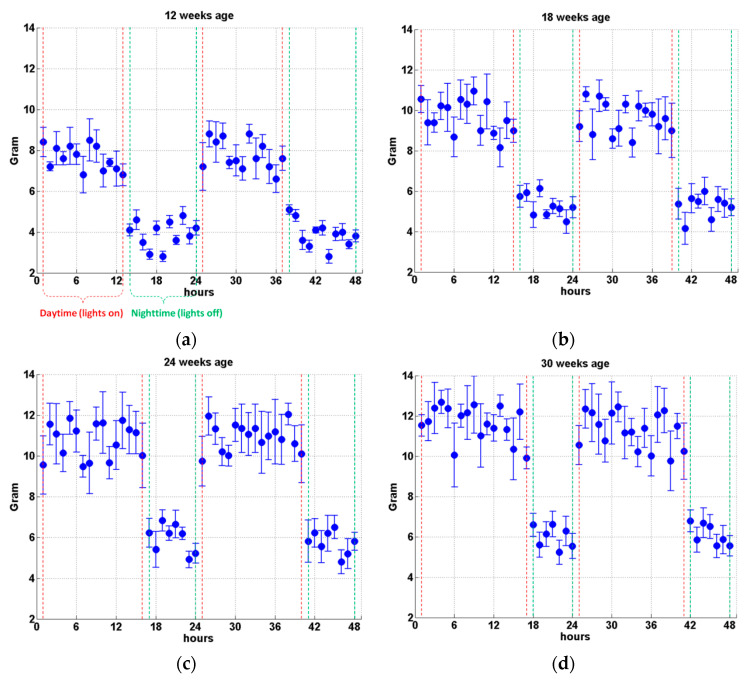
Manure weight measured (per hour per hen) at four typical laying hen ages of (**a**) 12 weeks, (**b**) 18 weeks, (**c**) 24 weeks, and (**d**) 30 weeks. Average value is provided with the standard deviation (error bar). Each test starts at 5 am (lights on) and lasts for 48 h with a measurement interval of one hour. Data between red-dash lines are measured in the daytime (lights on), and data between green-dash lines are recorded in the nighttime (lights off).

The hourly increment of manure weight was measured and calculated during each test from the laying hen 10 weeks age to 30 weeks age. The results from four typical ages are presented here in the format of the mean value (M) and standard deviation (ST). As it can be clearly seen in Figure 5, the manure produced by the birds every hour in the daytime (lights on) is apparently more than that in the nighttime (lights off). For 12 weeks age, the average hourly increment of manure weight recorded in the daytime is approximately 7.6 g per hour per hen, while the value is about 3.9 g per hour per hen in the nighttime (see Figure 5a). Furthermore, due to the increase in the amount of feed in the following weeks, the birds produce more manure every hour than that in 12 weeks age. The corresponding average hourly increment of manure weight in the daytime for 18, 24, and 30 weeks age is about 9.2, 11.1, and 11.8 g per hour per hen, respectively. Meanwhile, the corresponding average hourly increment of manure weight recorded in the nighttime for 18, 24, and 30 weeks age is about 5.4, 5.8, and 6.2 g per hour per hen, respectively (see Figure 5).

Although the day length increases gradually from the laying hen 10 weeks age to 30 weeks age (see Table 2) as the laying hens enter the laying period from the rearing period, the recorded feed to manure ratio is kept at around 2.04 in each week as it can be seen in Table 3. Detailed information of average hourly increment of manure weight measured in the daytime and nighttime in each week is also provided in Table 3. In addition, the recorded weekly moisture content (MC) ranges from 72.7%±4.0% to 82.3%±2.1%, and there is no apparent trend or pattern detected. However, the measured manure PH value demonstrated a downward trend from the beginning of the experiment to the end. The maximum value of PH=7.9±0.3 is recorded in the 11 weeks age, while the minimum value of PH=6.8±0.1 is measured in the 28 weeks age. It is hypothesized that the changes in the content of feed and the climate might be responsible for the PH decrease, and further study is required to provide solid conclusions.

### 3.2. Manure Coverage Proportion (MCP) and Area

**Figure 6:**
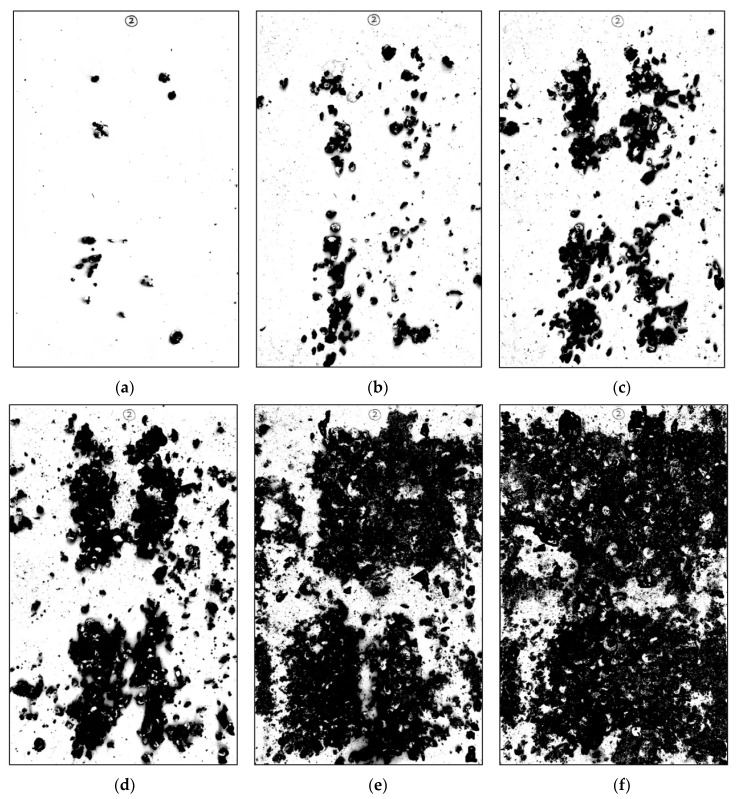
Binary images of the manure on one of the pp plates for the 24 weeks age. Photographs taken at the (**a**) 1st hour, (**b**) 4th hour, (**c**) 8th hour, (**d**) 12th hour, (**e**) 30th hour, and (**f**) 44th hour.

Figure 6 illustrates the binary images of the manure on one of the pp plates taken at different times during the experiment for 24 weeks age. The pictures indicate the corresponding manure coverage proportion at the 1st, 4th, 8th, 12th, 30th, and 44th hour is approximately 1.65%, 11.42%, 23.77%, 38.93%, 67.12%, and 81.26%, respectively. Meanwhile, the corresponding projected manure area is calculated to be $0.01{\;m}^{2}$, $0.067{\;m}^{2}$, $0.139{\;m}^{2}$, $0.227{\;m}^{2}$, $0.393{\;m}^{2},$ and $0.475{\;m}^{2},$ respectively. Finally, the total manure area in the poultry house ($A_{T}$) could then be estimated using the following equation:

(1) $$A_{T}={\mathrm{CP}}_{i}\times A_{\mathrm{plate}}\times N_{\mathrm{cage}}$$

where ${\mathrm{CP}}_{i}$ is the manure coverage proportion at the $i^{\mathrm{th}}$ hour after the most recent manure removal, $A_{\mathrm{plate}}$ is the pp plate area, which is roughly equal to the cage area, and $N_{\mathrm{cage}}$ is the total number of cages in the house.

By observing the photographs of the manure, a clear message can be read that with the increase in coverage proportion, the phenomenon of manure overlap becomes apparent. It is extremely difficult to exactly measure the surface area of the manure due to its irregular shape, but the projected manure area still represents a suitable method to approximate the surface area since the release of ammonia from manure is significantly affected by the airflow characteristics (including temperature, velocity, turbulence, etc.) above the release surface according to previous studies [14]. Therefore, manure covered underneath has a limited contribution to the total ${\mathrm{NH}}_{3}$ emissions and the projected area would not be considerably different from the true surface area since the height of the overlap is not large according to the field observation.

**Figure 7:**
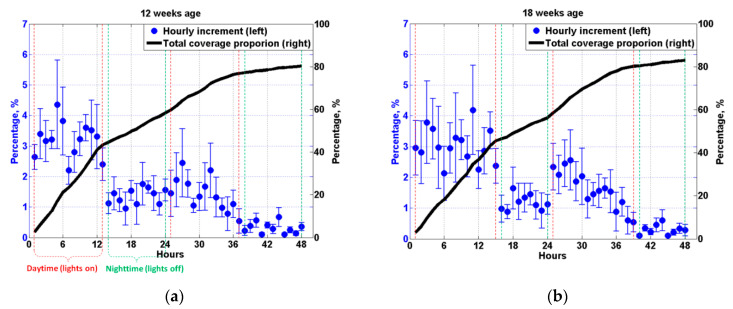

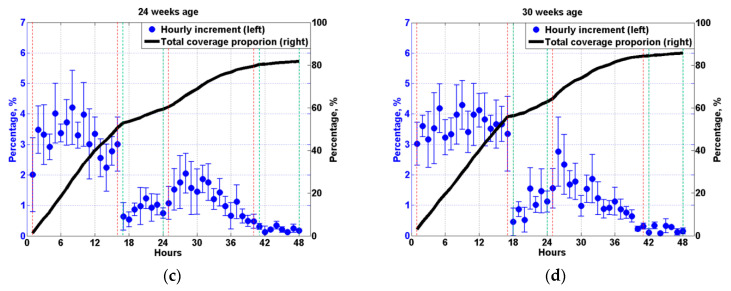
Hourly increment of manure coverage proportion (left *Y*-axis) and total coverage proportion (right *Y*-axis) on the pp plates measured in (**a**) 12 weeks age, (**b**) 18 weeks age, (**c**) 24 weeks age, and (**d**) 30 weeks age. Each test starts at 5 am (lights on) and lasts for 48 h with a measurement interval of one hour. Data between red-dash lines are measured at daytime (lights on), and data between green-dash lines are measured at nighttime (lights off).

Figure 7 further shows the hourly increment of manure coverage proportion (MCP) on the plates measured at four typical laying hen ages. The result is presented in the format of the mean value calculated from six plates with standard deviation (error bar). As it can be seen in Figure 7a, the hourly increment of MCP for the first daytime (from the 1st hour to the 13th hour) is approximately 3.34% per hour. Lights were turned off from the 14th hour for 12 weeks age, and an apparent decrease in hourly increment is recorded in the first nighttime (from the 14th hour to the 24th hour) with a mean value of about 1.35% per hour, which agrees with the decrease in hourly increment of manure weight measured in the nighttime as it can be seen in Figure 5a and Table 3. The total MCP after the first day (the 24th hour) is calculated to be 58.33%, as indicated by the solid black line (right *Y*-axis) in Figure 7a. For the second daytime, the hourly increment of MCP increases at the beginning from the 25th hour to about the 32nd hour due to the feeding activity. However, because of the aggravation of manure overlap, the hourly increment of MCP demonstrates a decreasing trend from the 33rd hour to the 37th hour. The overall mean value of hourly increment of MCP for the second daytime (from the 25th hour to the 37th hour) is measured to be only about 1.43% per hour, which is significantly lower than that in the first daytime although the manure weight dropped by the birds during the second daytime is roughly equal to that during the first daytime as demonstrated in Figure 5a. Because of limited manure dropped by the birds during nighttime and severe manure overlap resulting from the existing large coverage proportion, the hourly increment of MCP measured for the second nighttime (from the 38th hour to the 48th hour) is very limited with an average value of merely 0.32% per hour. Finally, the total manure coverage proportion climbs to about 80.35% at the end of the experiment (the 48th hour).

As illustrated in Figure 7b, the data measured for laying hen of 18 weeks age shows a similar trend with that in 12 weeks age. The hourly increment of MCP is relatively large at the beginning of the experiment, with a mean value of 3.04% per hour for the first daytime (from the 1st hour to the 15th hour). The average hourly increment decreases to about 1.18% per hour for the first nighttime (from the 16th hour to the 24th hour) in accordance with the decrease in the hourly increment of manure weight (see Figure 5b). For the second daytime from the 25th hour to the 39th hour, the hourly increment of MCP rebounds to approximately 1.61% per hour, which is only about half of that for the first daytime due to the manure overlap. The total manure coverage proportion ends up at about 82.94%, with very limited hourly increments observed from the 40th hour to the 48th hour (the second nighttime).

**Figure 8:**
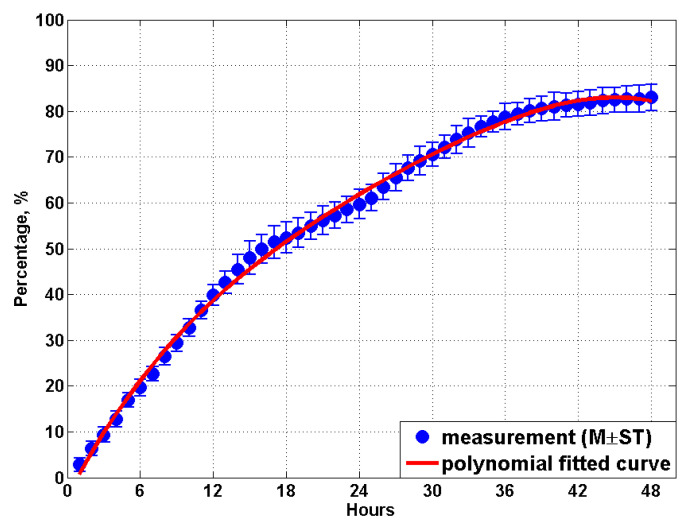
The measured manure coverage proportion curve with time and the polynomial fit curve within 48 h. Mean values (M) and standard deviations (ST) are calculated based on all 21 weeks’ data.

By examining the results measured in other weeks, for example, the MCP data for 24 and 30 weeks age as shown in Figure 7c,d, it is found that all the recorded coverage proportion curves (the solid black line) demonstrate a similar trend with four distinct stages: firstly, an almost linear relationship is detected between the MCP and the time (hours) with a gradient ranges from about 3.0% to 3.5%; secondly, for the first nighttime the curve slope reduces to about 0.9%~1.4%; thirdly, due to the manure overlap, the total coverage proportion curve during the second daytime only shows a moderate gradient ranges from 1.35% to 1.8%, which is considerably lower than the gradient at the beginning of the test. Finally, when the experiment enters into the second nighttime, the curve gradient reduces to only about 0.3%. The MCP data measured from all the 21 weeks are then averaged, and mean values are plotted in Figure 8. Results from the present study indicate the manure produced by the birds in one day would cover approximately 60% of the area of the manure belt, and more than 80% of the belt area would be covered within 48 h.

Furthermore, no significant correlation (r=0.11,p>0.05) can be found between the hourly increment of manure weight and the hourly increment of manure coverage proportion by examining the data recorded from 10 to 30 weeks age using Pearson’s correlation coefficient in SPSS. The insignificant correlation indicates that the hourly increment of MCP or manure area on the manure belt would not necessarily be affected by the variation of the amount of manure dropped by the birds.

A polynomial fitted curve is created to represent the total manure coverage proportion within 48 h after the most recent manure removal, as can be seen in Figure 8. The fitted curve shows suitable agreement ($R^{2}=0.997$) with the experimental measurements and falls within the standard error range at each hour. The equation of the fitted curve for predicting MCP reads

(2) $${\mathrm{MCP}}_{48}=P_{1}\times h^{4}+P_{2}\times h^{3}+P_{3}\times h^{2}+P_{4}\times h+P_{5}$$

where h is the time (hours) after the most recent manure removal and ${\mathrm{the}\;\mathrm{values}\;\mathrm{of}\;\mathrm{coefficients}\;\mathrm{of}\;P}_{1}\sim P_{5}$ are provided in Table 4.

**Figure 9:**
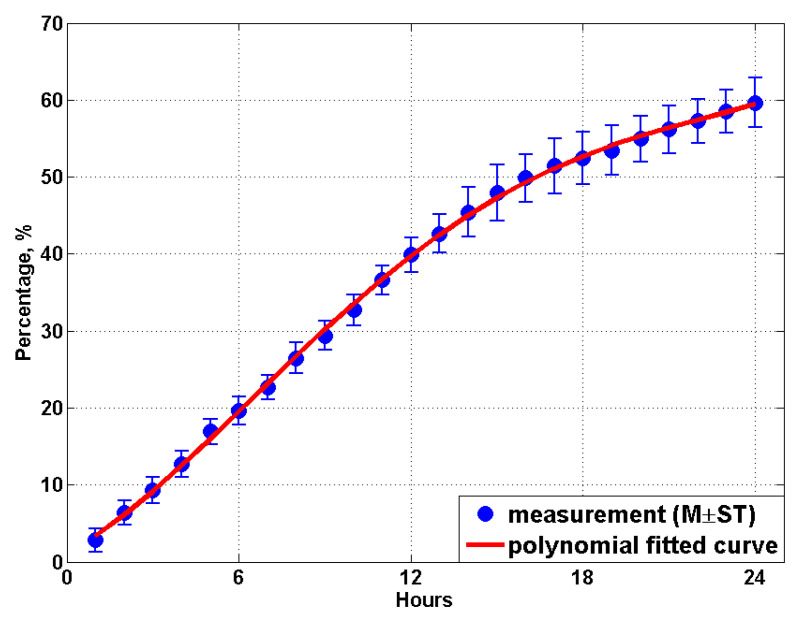
The measured manure coverage proportion with time and polynomial fit curve within 24 h. Mean values (M) and standard deviations (ST) are calculated based on all 21 weeks’ data.

In addition, for some poultry farms where the manure belt is cleared every 24 h, the equation for predicting MCP within 24 h is also provided and reads

(3) $${\mathrm{MCP}}_{24}=P_{6}\times h^{4}+P_{7}\times h^{3}+P_{8}\times h^{2}+P_{9}\times h+P_{10}$$

where ${\mathrm{the}\;\mathrm{values}\;\mathrm{of}\;\mathrm{coefficients}\;\mathrm{of}\;P}_{6}\sim P_{10}$ is provided in Table 4, and the polynomial fitted curve is shown in Figure 9, which demonstrates suitable agreement ($R^{2}=0.999$) with the data measured from the field tests.

## 4. Conclusions

In order to fill the gap of the understanding of the relationship between manure coverage proportion on the manure belt and time during poultry farming, experimental measurements were performed in a manure belt tunnel-ventilated layer house with a stocking density of about $14\;\mathrm{birds}/m^{2}$ using laying hens from 10 to 30 weeks age. In each week, the test began at 5 am in the morning and lasted for 48 h with a measurement interval of one hour. Six polypropylene (pp) plates were placed randomly above the manure belt to collect the manure dropped by the hens in order to provide average results. The manure weight was investigated every hour, and photographs of the pp plates were taken at the same time using a pre-fixed camera. Binary images were then produced based on the color pictures, and the objects coverage proportion was estimated, and the manure area was calculated at the same time. In addition, important manure parameters, including pH and moisture content (MC), were also measured every week to provide basic data for future studies.

Based on the experimental results from the present study, some conclusions can be drawn as follows:

1. The feed to manure ratio is kept at ~2.04 from the laying hen 10 weeks age to 30 weeks age;
2. The hourly increment of manure coverage proportion measured in different laying hen ages demonstrates similar trends and values with four distinct stages within 48 h;
3. For stocking density around $14\;\mathrm{birds}/m^{2}$, the manure coverage proportion on the manure belt at the 24th hour after the most recent manure removal is about 60%, while the value is approximately 82% at the 48th hour;
4. The statistical analyses demonstrate no significant correlation between the hourly increment of manure weight and the hourly increment of manure coverage proportion on the manure belt.

Finally, this study provides new knowledge and prediction models for estimating the hourly manure coverage proportion and area in the poultry house, which could be directly applied in thermodynamic models developed in the literature to predict the indoor hourly ammonia emissions achieving the goal of precision poultry farming.

## Figures and Tables

**Table 1:** Detailed information on the experimental measurements.

	Information	Notes
Experiment period	10 weeks age~30 weeks age	Measurements performed once a week
Measurement interval	1 h	Starts at 5 am and lasts for 48 h (2 days)
Number of pp plates	6	To calculate the hourly average values
Parameters concerned	Manure weight, coverage proportion, area	The resolution of the scale is 0.1 g, and the resolution of the photographs is 4032×3024 pixels
Staff involved	6 people	Rotating schedule

**Table 2:** Lighting schedule during experiment period and targeted weight of the hens.

Week	Daylength (Hours)	Targeted Weight (g)	Week	Daylength (Hours)	Targeted Weight (g)
10	13	1140	21	16	2130
11	13	1230	22	16	2220
12	13	1320	23	16	2300
13	14	1410	24	16	2380
14	14	1500	25	17	2460
15	14	1590	26	17	2540
16	14	1680	27	17	2630
17	15	1770	28	17	2700
18	15	1860	29	17	2770
19	15	1950	30	17	2840
20	15	2040			

**Table 3:** Manure data measured and recorded each week.

Age (Week)	Feed (g)	Average Hourly Increment of Manure Weight Measured In The Daytime (Gram per Hour per Hen)	Average Hourly Increment of Manure Weight Measured in the Nighttime (Gram per Hour per Hen)	Feed to Manure Ratio	PH (M±ST)	MC, % (M±ST)
10	60	7.0	3.7	2.19	7.5±0.2	73.3±3.9
11	63	7.2	3.9	2.16	7.9±0.3	71.5±2.8
12	67	7.6	3.9	2.12	7.6±0.3	78.7±1.6
13	70	8.0	4.5	2.24	7.3±0.2	79.3±1.1
14	74	8.2	4.6	2.17	7.1±0.1	76.1±1.9
15	78	8.4	4.8	2.11	7.5±0.1	74.5±3.5
16	83	8.5	5.1	2.05	7.6±0.3	81.6±2.9
17	88	8.9	5.0	2.03	7.3±0.1	75.4±1.8
18	93	9.2	5.4	2.01	7.7±0.3	74.5±3.4
19	98	9.6	5.3	1.95	6.9±0.3	78.9±2.3
20	103	10.1	5.6	1.96	7.3±0.2	81.2±1.7
21	108	10.6	5.8	1.97	7.1±0.2	77.4±3.9
22	110	10.9	5.5	2.01	7.0±0.2	72.7±4.0
23	112	11.0	5.9	1.93	7.3±0.1	77.3±3.3
24	114	11.1	5.8	1.94	7.1±0.3	79.1±1.1
25	116	11.4	6.0	1.95	7.4±0.3	72.9±1.5
26	118	11.6	6.1	2.01	7.2±0.2	82.3±2.1
27	120	11.8	6.0	2.02	7.1±0.2	74.9±2.9
28	120	11.7	6.2	2.01	6.8±0.1	75.8±1.5
29	120	11.9	6.1	2.04	6.9±0.1	76.8±2.4
30	120	11.8	6.2	2.03	7.0±0.2	79.2±2.6

Note: manure PH and MC are provided in the format of mean value (M) ± standard deviation (ST).

**Table 4:** Coefficients for polynomial fitted curves.

Coefficient	Value	Coefficient	Value
$P_{1}$	$-3.359\times 10^{-5}$	$P_{6}$	$3.234\times 10^{-4}$
$P_{2}$	$3.621\times 10^{-3}$	$P_{7}$	$-1.861\times 10^{-2}$
$P_{3}$	−0.1648	$P_{8}$	0.2786
$P_{4}$	5.081	$P_{9}$	1.992
$P_{5}$	−4.105	$P_{10}$	1.147

## Data Availability

Not applicable.
